# Comparison of Clinical Factors Between Recurrent and Non-recurrent Atrial Fibrillation Within One Year After Catheter Ablation by the Same Operators at a Regional Core Hospital in Japan

**DOI:** 10.7759/cureus.93365

**Published:** 2025-09-27

**Authors:** Toshio Katagiri, Takuya Kishi, Yoshitaka Hirooka, Tadashi Yamamoto, Masataka Kajiwara, Takashi Fujimura, Tomohiro Imamura, Ayako Takamori, Tomohito Inage

**Affiliations:** 1 Cardiology, International University of Health and Welfare Graduate School of Medicine, Okawa, JPN; 2 Cardiology, Kouhou-kai Takagi Hospital, Okawa, JPN; 3 Clinical Research Center, Saga University Hospital, Saga, JPN

**Keywords:** atrial fibrillation, catheter ablation, enlarged left atrium, recurrence, renal function

## Abstract

Although catheter ablation has emerged as a cornerstone treatment for atrial fibrillation, recurrence after catheter ablation occurs in approximately 20%-40% of patients within one year. Moreover, advances in devices, evolving treatment strategies, and differences in operator proficiency are believed to significantly impact outcomes. Whether results from large-scale international clinical trials can be directly applied to clinical practice at a regional core hospital in Japan remains a matter of debate. Considering these backgrounds, this study aimed to compare the clinical factors between recurrent and non-recurrent groups after atrial fibrillation catheter ablation in a regional core hospital in Japan, using the same treatment approach conducted by the same operators. A total of 368 consecutive patients who had their first catheter ablation for atrial fibrillation at our hospital between January 2017 and March 2022 were divided into two groups: non-recurrent (n = 311) and recurrent (n = 57). The recurrent group had significantly longer procedure time (196 ± 64 vs. 174 ± 66 minutes; 95% confidence interval (CI) -0.05, 0.62; P = 0.021), more cases of early recurrence (42% vs. 13%; 95% CI 2.69, 8.82; P = 0.001), higher rates of long-standing persistent atrial fibrillation (39% vs. 22%; 95% CI 1.23, 4.08; P = 0.008), greater left atrial volume index (LAVI) (49.8 ± 19.7 vs. 37.7 ± 14.6 mL/m^2^; 95% CI 6.46, 17.56; P = 0.001), more patients with LAVI > 48.9 mL/m^2^ (51% vs. 22%; 95%CI 2.01, 6.83; P = 0.0001), and more patients with the estimated glomerular filtration rate (eGFR) < 45 mL/min/1.73 m^2 ^(26% vs. 17%; 95% CI 0.98, 3.50; P = 0.046) than the non-recurrent group. Additional left atrial posterior wall isolation (LAPWI) for persistent and long-lasting persistent atrial fibrillation and cavotricuspid isthmus (CTI) ablation for long-lasting persistent atrial fibrillation were more frequent in the recurrent group (LAPWI for persistent atrial fibrillation (26% vs. 14%; 95% CI 1.14, 4.36; P = 0.029), LAPWI for long-lasting persistent atrial fibrillation (19% vs. 8%; 95% CI 1.21, 5.67; P = 0.023), CTI ablation for long-lasting persistent atrial fibrillation (30% vs. 13% cases; 95% CI 1.53, 5.11; P = 0.001)). Recurrent rate was significantly lower in cryoballoon than in radiofrequency catheter ablation in all types of atrial fibrillation. In the non-recurrent group, brain natriuretic peptide (BNP) and LAVI were significantly lower, and left ventricular ejection fraction (LVEF) was significantly higher at one year after catheter ablation than before (BNP: 63.5 ± 138.4 vs. 144.8 ± 168.3 pg/mL, P = 0.001, 95% CI -0.69, -0.37; LAVI: 31.3 ± 12.3vs. 37.1 ± 14.6 mL/m^2^, P = 0.001, 95% CI -0.69, -0.32; LVEF: 58.9 ± 7.7 vs. 55.2 ± 10.2 %, P = 0.001, 95% CI -0.25, -0.57). In the recurrent group, eGFR was significantly lower at one year after catheter ablation than before catheter ablation (53.3 ± 25.2 vs. 60.4 ± 24.1 mL/min/1.73 m^2^, P = 0.021, 95% CI -0.58, -0.05). In conclusion, in cases of catheter ablation for atrial fibrillation with significantly large LAVI (>49.8 mL/m^2^), eGFR < 45 mL/min/1.73 m^2^, extended procedure time, and early recurrence within 90 days after catheter ablation, recognizing the possibility of recurrence within one year after catheter ablation and conducting follow-up with a focus on renal protection may provide valuable insights for catheter ablation of atrial fibrillation in regional core hospitals in Japan.

## Introduction

Catheter ablation has emerged as a cornerstone treatment for atrial fibrillation, particularly in patients with symptomatic and drug-refractory atrial fibrillation. According to multiple randomized controlled trials, its efficacy in achieving rhythm control surpasses anti-arrhythmic drug therapy. For patients with paroxysmal atrial fibrillation, catheter ablation offers a high rate of freedom from atrial fibrillation recurrence, with success rates of approximately 70% for paroxysmal atrial fibrillation and 50% for persistent atrial fibrillation [1,2]. In persistent atrial fibrillation, catheter ablation is also superior to medical therapy. A meta-analysis of 46 studies involving 3,819 patients showed that catheter ablation significantly reduces the risk of recurrent atrial fibrillation compared to anti-arrhythmic drug therapy (odds ratio (OR) 0.32; 95% confidence interval (CI) 0.20, 0.53; P < 0.001) [3]. Pulmonary vein isolation (PVI) remains the cornerstone of the procedure, with adjunctive linear ablation within the left atrium further improving outcomes [3]. Atrial fibrillation burden and symptomatic episodes were reduced over five years post-ablation [4]. Importantly, catheter ablation has demonstrated benefits beyond rhythm control. For patients with concomitant heart failure, catheter ablation reduces hospitalization and mortality compared to medical therapy [5]. These findings underscore its role not only in rhythm management but also in improving overall cardiovascular outcomes.

Despite its efficacy, recurrence of atrial fibrillation after catheter ablation occurs in approximately 20%-40% of patients within one year [5-7]. The primary mechanism for recurrence is pulmonary vein reconnection, necessitating repeat ablation procedures in cases where symptoms persist. Beyond technical aspects, several clinical factors influence recurrence rates. Key risk factors for atrial fibrillation recurrence include advanced age, long-standing persistent atrial fibrillation, and structural cardiac changes such as left atrial enlargement or fibrosis. Comorbidities like hypertension, obesity, obstructive sleep apnea, and metabolic syndrome are also strongly associated with higher recurrence rates [6,7]. Addressing these modifiable risk factors through lifestyle interventions, such as weight loss, blood pressure control, and treatment for obstructive sleep apnea, has significantly improved outcomes [7]. The duration of atrial fibrillation before ablation is another critical determinant. Patients with paroxysmal atrial fibrillation have better outcomes compared to those with persistent or long-standing persistent atrial fibrillation due to less extensive atrial remodeling [8]. Additionally, systemic conditions like diabetes mellitus and thyroid dysfunction exacerbate pro-arrhythmic substrates and increase recurrence risk [6,8]. Repeat ablation is often required for recurrent arrhythmias such as atrial tachycardia or flutter due to incomplete lesion sets or gaps in prior ablations [1]. While repeat procedures achieve success rates comparable to initial ablations, addressing underlying risk factors remains pivotal for long-term rhythm control [6]. However, advances in devices, evolving treatment strategies, and differences in operator proficiency are believed to impact outcomes. Furthermore, it is debatable whether the results of large-scale international clinical trials can be directly applied to clinical practice at core hospitals in Japanese regions, where aging is severe and many patients have multiple comorbidities.

Considering these backgrounds, this study aimed to compare the clinical characteristics of recurrent and non-recurrent groups after atrial fibrillation catheter ablation, using the same treatment approach by the same group of practitioners, at our facility. Our hospital, where this study was conducted, is the only general hospital in a regional city (population 30,000) that faces severe aging issues. It is also the only medical institution capable of performing catheter ablation in a region with a population of approximately one million. Furthermore, the entire treatment process, from outpatient diagnosis to inpatient treatment and subsequent ongoing outpatient follow-up, is completed within our hospital. This characteristic allows us to manage follow-up for all patients who undergo catheter ablation at our facility. Given this background, our objective is not to explore generalizable risk factors but rather to analyze our own clinical practice. Furthermore, we aim to propose key treatment strategies and follow-up care considerations for catheter ablation in atrial fibrillation at our hospital and other regional core hospitals in Japan that face similar circumstances. The one-year recurrent rate was adopted because it captures the majority of recurrences (approximately 80%-90%). A six-month follow-up period has been associated with the potential to miss late recurrences or overestimate the actual treatment effect. Conversely, a one-year follow-up period may miss some late recurrences and inadequately assess long-term risk factors. Traditionally, a guideline recommended a one-year follow-up [9]. However, recent international guidelines recommend a two-year follow-up period [1,10]. We determined that a two-year analysis would not suit the objectives of this study because our facility changed its catheter ablation operator during that time.

## Materials and methods

This is a single-center, retrospective observational study. Inclusion criteria were as follows: patients undergoing initial catheter ablation for atrial fibrillation at Takagi Hospital between January 2017 and March 2022. Exclusion criteria were as follows: (1) the patient refuses to give informed consent, (2) the patient is a candidate for coronary bypass surgery or cardiac valve surgery, and (3) the patient has carcinoma with a prognosis of one year or less. Finally, 368 patients were included. Ablation was performed for atrial fibrillation documented by 12-lead electrocardiogram (ECG) or ECG monitoring, regardless of subjective symptoms or history of anti-arrhythmic drug use. Atrial fibrillation was classified into three categories based on its duration, in accordance with the Japanese clinical practice guideline [11]. Paroxysmal atrial fibrillation is defined as atrial fibrillation lasting for <7 days. If the patient is defibrillated within seven days, it is also classified as paroxysmal atrial fibrillation. Persistent atrial fibrillation is defined as atrial fibrillation lasting more than seven days after occurrence. It includes cases of defibrillation after seven days with an anti-arrhythmic drug or direct current cardioversion. Long-standing persistent atrial fibrillation is defined as atrial fibrillation persisting for >1 year [9-11]. Before ablation, oral anticoagulant therapy was applied for more than three weeks preoperatively, and the absence of a thrombus in the left atrium was confirmed by contrast-enhanced CT or transesophageal echocardiography. Exclusion criteria for catheter ablation in the present study were patients with suspected thrombosis in the left atrium, first-episode paroxysmal atrial fibrillation, or contraindication to anticoagulant therapy. To explain briefly, three-dimensional mapping of the left atrium was performed using a CARTO3 (Biosense Webster, part of Johnson & Johnson MedTech, Irvine, CA, USA) mapping system or Ensite Precision/Velocity (Abbott, St. Paul, MN, USA) mapping system with a multipolar mapping catheter (PENTRAY, Biosense Webster, Inc., Diamond Bar, CA, USA or H.D. Grid, Abbott, St. Paul, MN, USA). A temperature monitoring (Esophaster, Japan Lifeline, Tokyo, Japan) at the level of the left atrium tagged the esophageal location and provided temperature feedback during the procedure.

Pulmonary vein isolation (PVI) was performed using contact force-sensing open irrigated catheters by radiofrequency catheter ablation (RFCA) or cryoballoon ablation using a 28 mm cryoballoon (Arctic Front Advance, Medtronic Inc, Minneapolis, MN, USA). In the case of paroxysmal atrial fibrillation, additional procedures were performed after PVI at the operator's discretion. For persistent and long-term atrial fibrillation, PVI was performed first. Subsequent additional procedures, such as left atrial posterior wall isolation (LAPWI) or tricuspid annulus-inferior vena cava isthmus (CTI) ablation, were left to the discretion of the two operators in this study. LAPWI was performed after PVI with a roof line connecting the superior aspect of the superior pulmonary vein and a floor line joining the most inferior aspect of the inferior pulmonary vein with the electrical isolation of the posterior wall of the left atrium as the endpoint. Cryoballoon ablation was mainly applied for paroxysmal atrial fibrillation without left atrial enlargement. In cases with evidence of non-PV foci, the ablation strategy involved the elimination of these foci. Superior vena cava (SVC) isolation was performed if arrhythmogenicity was detected in the SVC. In this study, catheter ablation was performed in all cases by two operators, with each serving as the primary operator and assistant in half of the cases. Decisions regarding procedure selection and additional procedures were made by mutual agreement between the two operators. Both operators had nearly equivalent experience in catheter ablation cases and were certified specialists by the Japanese Circulation Society and certified catheter ablation operators by the Japanese Arrhythmia and Electrocardiology Society.

Anti-arrhythmic drugs were recommended to be discontinued after the blanking period (90 days post-ablation). Oral anticoagulants were mandatory for at least three months after ablation. Echocardiography was performed 12 months after the procedure, and 24-hour Holter monitoring was performed 3, 6, and 12 months after the procedure. A 12-lead ECG was recorded as appropriate when symptoms appeared. Recurrence was defined as atrial fibrillation, atrial tachycardia, or atrial flutter lasting more than 30 seconds after the blanking period. Recurrence within three months was defined as early recurrence when additional ablation was not performed [1,9-11]. Recurrence after ablation occurs most frequently during the first year following the 90-day blanking period, making the one-year follow-up period the most critical timeframe for determining treatment success [11]. Furthermore, international expert consensus statements explicitly recommend 12 months as the standard follow-up period for reporting ablation success rates [1].

Gender, age, weight, height, body mass index, types of atrial fibrillation, procedure time, fluoroscopy time, and comorbidity were recorded at ablation in the electronic medical record. Early recurrence, procedure details, and procedural complications were compared between the non-recurrent and recurrent groups. Brain natriuretic peptide (BNP), left ventricular ejection fraction (LVEF), left atrial volume index (LAVI), and estimated glomerular filtration rate (eGFR) were evaluated before and one year after the procedure. There was no missing data for the items analyzed in the databases used. The study was conducted in accordance with the Declaration of Helsinki and received ethical approval from the Ethical Committee of Takagi Hospital (KR079) and the Ethical Committee of the International University of Health and Welfare (22-IFH-031). Informed consent was obtained from all patients who met the inclusion criteria, permitting the use of their data for this research. All patients were receiving ongoing outpatient care at our hospital, and none had died or were in a state where their consent could not be confirmed.

Continuous variables were assessed for normality using the Shapiro-Wilk test and visual inspection of histograms and Q-Q plots. Normally distributed continuous variables were compared using Student's t-test for two groups or one-way analysis of variance for multiple groups, presented as means ± standard deviations. Non-normally distributed continuous variables were analyzed using the Mann-Whitney U test for two groups or the Kruskal-Wallis test for multiple groups, presented as medians with interquartile ranges. Categorical variables were compared using the chi-square test or Fisher's exact test when expected cell counts were less than five. For comparisons of continuous variables between groups, we calculated odds ratios (ORs), and for categorical variables, we calculated effect sizes (Cohen's d). Both were determined along with their 95% CIs, and statistical power was also calculated. Recurrent rates were compared across atrial fibrillation types using the Kruskal-Wallis test with post-hoc analysis. Results were presented as frequencies and percentages. JMP Pro 16 (SAS Institute Inc., Cary, NC, USA) was used for all analyses, and statistical significance was defined as P < 0.05.

## Results

Table 1 shows the characteristics of the non-recurrent (311 cases) and recurrent (57 cases) groups within one year after catheter ablation for atrial fibrillation patients from January 2017 to March 2022. The timing of recurrence was as follows: 38 cases at 9 months after catheter ablation, 13 cases at 12 months, 2 cases at 6 months, and 4 cases at 3 months (early recurrence persisting thereafter). Of the 64 cases with early recurrence within 90 days after catheter ablation, 40 cases spontaneously recovered to sinus rhythm 3 months after catheter ablation and remained recurrence-free thereafter. Twenty cases spontaneously recovered to sinus rhythm 3 months after catheter ablation but subsequently experienced recurrence (1 case at 6 months, 17 cases at 9 months, 2 cases at 12 months), and 4 cases had persistent recurrence at 3 months. The recurrent group (n = 57) had longer procedure times and more cases of early recurrence than the non-recurrent group (n = 311). The mean age tended to be higher in the recurrent group, but ORs and statistical power were low, and no significant difference was observed (72.8 ± 10.1 vs. 70.2 ± 10.2 years, OR 1.31, 95% CI 0.71-2.41, power 18%). In the age-specific analysis, the non-recurrent group was more common among those under 65 years old; however, the ORs and statistical power were low, and no significant difference was observed. Among those aged 65 or 75 years or older, the recurrent group tended to be more common, and while the OR was greater than 1, statistical power was low, and no significant difference was observed. Home blood pressure and comorbidities were similar between the non-recurrent and recurrent groups. The average heart rate during atrial fibrillation episodes was similar between the recurrent and non-recurrent groups. The recurrent group had significantly longer procedure time (196 ± 64 vs. 174 ± 66 minutes, P = 0.021) and more cases of early recurrence (24 (42%) vs. 40 cases (13%), P = 0.001).

**Table 1:** The characteristics of patients with successful catheter ablation for atrial fibrillation during January 2017 to March 2022 Data are mean ± SD. BMI: body mass index, BP: blood pressure, TIA: transient ischemic attack, OR: odds ratio, CI: confidence interval. *P < 0.05, **P < 0.01; compared between non-recurrent and recurrent groups.

	Non-recurrent (n = 311)	Recurrent (n = 57)	OR or Cohen's d	95% CI	Power	P
Males	212 (68%)	42 (74%)	1.31	0.71, 2.41	18%	0.391
Age (year)	70.2 ± 10.2	72.8 ± 10.1	0.26	-0.03, 0.54	42%	0.077
<65	95 (31%)	12 (22%)	0.61	0.31, 1.20	31%	0.417
65-75	117 (38%)	23 (40%)	1.12	0.63, 2.00	7%	0.696
>75	99 (32%)	22 (38%)	1.35	0.75, 2.41	17%	0.318
Body weight (kg)	61.1 ± 12.5	62.4 ± 12.2	0.10	-0.18, 0.39	11%	0.469
Height (cm)	161.9 ± 9.8	162.5 ± 10.3	0.06	-0.22, 0.34	7%	0.674
BMI (kg/m^2^)	23.2 ± 3.5	23.4 ± 3.9	0.06	-0.23, 0.34	7%	0.697
Home BP (mmHg)	138 ± 13	141± 12	0.24	-0.02, 0.49	45%	0.063
Procedure time (min)	174 ± 66	196 ± 64	0.34	0.05, 0.62	64%	0.021^*^
Fluoroscopy time (min)	29 ± 18	27 ± 14	-0.11	-0.39, 0.18	11%	0.464
Early recurrence	40 (13%)	24 (42%)	4.90	2.69, 8..92	99%	0.0001^**^
Comorbidity						
Congestive heart failure	82 (26%)	15 (26%)	0.72	0.39, 1.33	49%	0.657
Hypertension	175 (56%)	31 (54%)	0.93	0.55, 1.57	6%	0.776
Diabetes mellitus	63 (20%)	13 (23%)	0.88	0.45, 1.73	40%	0.652
Stroke/TIA	48 (15%)	12 (21%)	1.47	0.72, 2.99	22%	0.293
Vascular disease	33 (11%)	8 (14%)	1.38	0.61, 3.12	22%	0.445
Heavy alcohol drinker	8 (3%)	2 (4%)	1.38	0.27, 6.94	6%	0.695

Table 2 shows the type of atrial fibrillation in the non-recurrent and recurrent groups. The Kruskal-Wallis test showed a significant difference in the types of atrial fibrillation in the recurrence group (H = 9.21, P = 0.012). Subsequent post-hoc tests revealed that long-standing persistent atrial fibrillation had a significantly higher rank compared to paroxysmal and persistent atrial fibrillation. Long-lasting persistent atrial fibrillation was more frequent in the recurrent group than in the non-recurrent group (22 (39%) vs. 68 (22%) cases; P = 0.008; OR 2.25; 95% CI 1.23, 4.08; statistical power 85%). Recurrent rate was significantly lower in cryoballoon than in RFCA (9% vs. 18%, P = 0.024) and in paroxysmal (10% vs. 15%, P = 0.031), persistent (6% vs. 13%, P = 0.037), and long-lasting persistent (17% vs. 25%, P = 0.018) atrial fibrillation. RFCA was more frequently performed for long-lasting persistent atrial fibrillation in the recurrent group than in the non-recurrent group (P = 0.027; OR 2.30; 95% CI 1.25, 4.21; statistical power 60%). Additional LAPWI was also used more often in the recurrent group than in the non-recurrent group for persistent atrial fibrillation (P = 0.029; OR 2.23; 95% CI 1.14, 4.36; statistical power 72%) and long-lasting persistent atrial fibrillation (P = 0.003; OR 2.62; 95% CI 1.21, 5.67; statistical power 89%). Furthermore, additional CTI ablation was significantly more common in the recurrent group than in the non-recurrent group for long-lasting persistent atrial fibrillation (P = 0.001; OR 3.12; 95% CI 1.53, 5.11; statistical power 95%). 

**Table 2:** Types of atrial fibrillation and procedures of catheter ablation in the non-recurrent and recurrent groups RFCA: radiofrequency catheter ablation, LAPWI: left atrial posterior wall isolation, CTI: cavotricuspid isthmus, CI: confidence interval. ^*^P < 0.05, ^**^P < 0.01; compared between the non-recurrent and recurrent groups. ^#^P < 0.05; compared to paroxysmal and persistent atrial fibrillation.

Types of atrial fibrillation and procedures	Non-recurrent (n = 311)	Recurrent (n = 57)	Odds ratio	95% CI	Power	P
Paroxysmal	166 (54%)	25 (44%)	0.68	0.39, 1.20	35%	0.172
RFCA	100	18	0.97	0.53, 1.79	5%	0.991
Cryoballoon	66	7	0.52	0.23, 1.20	27%	0.175
Additional LAPWI	69	11	0.84	0.41, 1.71	6%	0.756
Additional CTI ablation	6	1	0.90	0.11, 7.54	5%	0.907
Persistent	77 (25%)	10 (18%)	0.65	0.31, 1.34	25%	0.242
RFCA	60	9	0.78	0.37, 1,69	9%	0.537
Cryoballoon	17	1	0.31	0.04, 2.37	12%	0.283
Additional LAPWI	43	15	2.23	1.14, 4.36	72%	0.029*
Additional CTI ablation	18	3	0.90	0.26, 3.07	6%	0.893
Long-standing persistent	68 (22%)	22 (39%)^#^	2.25	1.23, 4.08	85%	0.008^**^
RFCA	63	21	2.30	1.25, 4.21	60%	0.027^*^
Cryoballoon	5	1	1.09	0.13, 9.53	5%	1
Additional LAPWI	26	11	2.62	1.21, 5.67	89%	0.023^*^
Additional CTI ablation	41	17	3.12	1.53, 5.11	95%	0.001^**^

Table 3 shows the selected treatment methods and adverse events of catheter ablation compared between the non-recurrent and recurrent groups within one year. The usage of RFCA and cryoballoon was similar between the non-recurrent and recurrent groups. The recurrent group had a significantly higher rate of additional LAPWI (P = 0.003; OR 2.32; 95% CI 1.32, 4.07; statistical power 88%) and additional CTI ablation (P = 0.013; OR 2.21; 95% CI 1.20, 4.06; statistical power 75%). Adverse events associated with catheter ablation (cardiac tamponade, pericardial effusion, transient ischemic attack, ST elevation on ECG, pulmonary vein stenosis, vascular complication, phrenic nerve paralysis, gastroparesis, and pericarditis) within one year after catheter ablation were similar between the non-recurrent and recurrent groups. 

**Table 3:** Selected catheter ablation methods and adverse events associated with catheter ablation compared between non-recurrent and recurrent groups RFCA: radiofrequency catheter ablation, LAPWI: left atrial posterior wall isolation, SVCI: superior vena cava isolation, PV: pulmonary vein, CTI: cavotricuspid isthmus, TIA: transient ischemic attack, ECG: electrocardiography, CI: confidence interval. *P < 0.05, **P < 0.01; compared between non-recurrent and recurrent groups.

	Non-recurrent (n = 311)	Recurrent (n = 57)	Odds ratio	95% CI	Power	P
RFCA	223 (72%)	48 (84%)	2.10	0.99, 4.47	43%	0.071
Cryoballoon	88 (28%)	9 (16%)	0.48	0.22, 1.01	43%	0.071
Additional procedure						
LAPWI	138 (44%)	37 (65%)	2.32	1.32, 4.07	88%	0.003^**^
SVCI	33 (11%)	4 (7%)	0.64	0.22, 1.84	16%	0.411
Non-PV foci ablation	15 (5%)	2 (4%)	0.72	0.16, 3.22	8%	0.664
CTI	65 (21%)	21 (37%)	2.21	1.20, 4.06	75%	0.013^*^
Mitral isthmus ablation	8 (3%)	2 (4%)	1.38	0.28, 6.81	6%	0.691
Adverse events						
Cardiac tamponade	4	1				
Pericardial effusion	5	1				
TIA	1	0				
ST elevation on ECG	4	0				
Pulmonary vein stenosis	0	0				
Vascular complication	2	1				
Phrenic nerve paralysis	2	0				
Gastroparesis	2	0				
Pericarditis	0	1				
Total	20 (6%)	4 (7%)	1.10	0.36, 3.36	6%	0.872

Table 4 shows BNP, LVEF, LAVI, and eGFR before catheter ablation in the recurrent and non-recurrent groups. LAVI was significantly greater in the recurrent group than in the non-recurrent group (49.8 ± 19.7 vs. 37.7 ± 14.6 mL/m^2^; P = 0.0001; Cohen's d = -0.78; 95% CI 6.46, 17.56; statistical power 99%). BNP, LVEF, and eGFR were similar between the recurrent and non-recurrent groups. The median value of 165.7 was set as the cutoff value for BNP between the recurrent and the non-recurrent groups. Sensitivity was 46% and specificity was 55%, indicating low classification accuracy. The group with values above this cutoff included 26 recurrent cases (46%) compared to 141 non-recurrent cases (45%). The OR was 1.44, with a 95% CI of 0.80, 2.58 and a P-value of 0.88, indicating no significance. To evaluate LAVI as a binary predictor for recurrence, an optimal cutoff value was determined to be 48.9 based on the receiver operating characteristic curves. This cutoff value yielded a sensitivity of 52% and a specificity of 78%. The recurrent group had significantly more patients with a LAVI > 48.9 than the non-recurrent group (29 (51%) cases vs. 68 (22%) cases; OR 3.63; 95% CI 2.01, 6.83; power 99%; P = 0.0001). Moreover, the recurrent group had more patients with eGFR < 45 mL/min/1.73 m^2^ than the non-recurrent group (P = 0.046; OR 1.85; 95% CI 0.98, 3.50; statistical power 52%).

**Table 4:** Comparison of BNP, LVEF, LAVI, and eGFR before catheter ablation between the non-recurrent and recurrent groups Data are mean ± SD. BNP: brain natriuretic peptide, LVEF: left ventricular ejection fraction, LAVI: left atrial volume index, eGFR: estimated glomerular filtration rate, OR: odds ratio, CI: confidence interval. *P < 0.05, **P < 0.01; compared between the non-recurrent and recurrent groups.

	No recurrent (n = 311)	Recurrent (n = 57)	OR or Cohen's d	95% CI	Power	P
BNP (pg/mL)	144.8 ± 168.2	186.5 ± 198.9	0.24	-7.39, 90.39	38%	0.141
>165.7	141 (45%)	26 (46%)	1.44	0.80, 2.58	53%	0.088
LVEF (%)	55.2 ± 10.2	54.4 ± 8.5	-0.08	-0.36, 0.20	9%	0.529
<40	17 (6%)	6 (11%)	2.03	0.79, 5.22	40%	0.088
40-50	68 (22%)	16 (28%)	1.39	0.74, 2.61	21%	0.301
>50	216 (70%)	39 (68%)	0.98	0.53, 1.83	5%	0.963
LAVI (mL/m^2^)	37.7 ± 14.6	49.8 ± 19.7	0.78	6.46, 17.56	99%	0.0001^**^
>48.9	68 (22%)	29 (51%)	3.63	2.01, 6.83	99%	0.0001^**^
eGFR (mL/min/1.73 m^2^)	64.5 ± 20.5	60.4 ± 24.1	0.2	-10.57, 2.37	25%	0.198
<60	128 (41%)	28 (49%)	1.25	0.71-2.19	16%	0.395
<45	53 (17%)	15 (26%)	1.85	0.98, 3.50	52%	0.046*

Table 5 shows BNP, LVEF, LAVI, and eGFR before and one year after catheter ablation in the non-recurrent and recurrent groups. In the non-recurrent group, BNP and LAVI were significantly lower, and LVEF was significantly higher at one year after catheter ablation than before catheter ablation (BNP (63.5 ± 138.4 pg/mL vs. 144.8 ± 168.3 pg/mL; P = 0.001; Cohen's d = -0.53; 95% CI -0.69, -0.37; statistical power 100%), LAVI (31.3 ± 12.3 mL/m^2 ^vs. 37.1 ± 14.6 mL/m^2;^ P = 0.001; Cohen's d = -0.47; 95% CI -0.69, -0.32; statistical power 100%), LVEF (58.9 ± 7.7% vs. 55.2 ± 10.2%; P = 0.001; Cohen's d = 0.41; 95% CI -0.25, -0.57; statistical power 99%)). No significant changes in heart failure symptoms (NYHA classification) were observed in either the recurrent group or the non-recurrent group before and after catheter ablation. In the recurrent group, eGFR was significantly lower at one year after catheter ablation than before catheter ablation (53.3 ± 25.2 mL/min/1.73 m^2^ vs. 60.4 ± 24.1 mL/min/1.73 m^2^; P = 0.021; Cohen's d = -0.31; 95% CI -0.58, -0.05; statistical power 64%).

**Table 5:** Changes in BNP, LVEF, LAVI, and eGFR before and one year after catheter ablation in the non-recurrent and recurrent groups Data are mean ± SD. BNP: brain natriuretic peptide, LVEF: left ventricular ejection fraction, LAVI: left atrial volume index, eGFR: estimated glomerular filtration rate, CI: confidence interval. *P < 0.05, **P < 0.01; compared before and one year after catheter ablation.

	Before ablation	One year after ablation	Cohen's d	95% CI	Power	P
BNP (pg/mL)						
Non-recurrent	144.8 ± 168.3	63.5 ± 138.4	-0.53	-0.69, -0.37	100%	0.001^**^
Recurrent	186.5 ± 198.9	126.0 ± 135.4	-0.14	-0.40, 0.13	17%	0.309
LVEF (%)						
Non-recurrent	55.2 ± 10.2	58.9 ± 7.7	0.41	0.25, 0,57	99%	0.001^**^
Recurrent	54.4 ± 8.5	56.2 ± 6.9	0.18	-0.08, 0.45	28%	0.172
LAVI (mL/m^2^)						
Non-recurrent	37.1 ± 14.6	31.3 ± 12.3	-0.47	-0.63, -0.32	100%	0.001^**^
Recurrent	49.8 ± 19.7	42.4 ± 15.2	-0.13	-0.39, 0.13	16%	0.344
eGFR (mL/min/1.73 m^2^)						
Non-recurrent	64.5 ± 20.5	64.8 ± 19.2	0.02	-0.14, 0.17	5%	0.851
Recurrent	60.4 ± 24.1	53.3 ± 25.2	-0.31	-0.58, -0.05	64%	0.021^*^

Between the non-recurrent (311 cases) and recurrent groups (57 cases), procedure time (P = 0.021, OR 4.90, power 64%), early recurrence (P = 0.0001, OR 4.90, power 99%), RFCA usage for long-lasting atrial fibrillation (P = 0.027, OR 2.30, power 60%), additional LVPWI (P = 0.003, OR 2.32, power 88%), additional CTI ablation (P = 0.013, OR 2.21, power 75%), LAVI (P = 0.001, Cohen's d = -0.74, power 99%), and frequency of eGFR < 45 (P = 0.046, OR 1.85, power 52%) showed significant differences in univariate analysis. All these factors are considered to be influenced by confounding factors such as age, severity of atrial fibrillation, left atrium fibrosis, different mapping systems, or procedural difficulty. Furthermore, for the non-recurrent group, adjusting for confounding factors among those showing significant differences in univariate analysis as risk factors for recurrence, four potential factors (early recurrence, RFCA usage, LAVI, eGFR < 45) were identified. To detect an OR of 1.5 in a logistic analysis with 80% power and 20 events per variable, the recurrent group requires at least 80 cases. We considered sensitivity analyses to evaluate the effects of different variable combinations and the categorization of continuous variables. However, we concluded that distinguishing clinical significance from statistical significance would become difficult. Therefore, we determined that multivariate analysis could not be performed correctly in this study. 

## Discussion

This retrospective study in a regional core hospital in Japan showed that (1) the recurrent group had significantly longer procedure time, more cases of early recurrence, higher rates of long-standing persistent atrial fibrillation, and greater LAVI than the non-recurrent group; (2) the recurrent rate was significantly higher in RFCA than in cryoballoon; (3) additional LAPWI for persistent and long-lasting persistent atrial fibrillation and CTI ablation for long-lasting persistent atrial fibrillation were more frequent in the recurrent group than in the non-recurrent group; (4) the non-recurrent group showed improved BNP, LAVI, and LVEF one year after the catheter ablation, and the recurrent group had more patients with LAVI > 48.9 mL/m^2^ or eGFR < 45 mL/min/1.73 m^2^ than the non-recurrent group; and (5) eGFR was significantly lower at one year after catheter ablation than before in the recurrent group, but not in the non-recurrent group.

The persistent existence of atrial fibrillation is acknowledged to lead to continuous electrical and structural remodeling and heightened resistance to treatment [12]. Bidirectional relationship between atrial fibrillation and atrial fibrosis, where persistent atrial fibrillation promotes continuous electrical and structural remodeling, ultimately leading to treatment resistance. Atrial fibrillation induces fibrosis through multiple interconnected processes, including stretch-induced fibroblast activation, oxidative stress, inflammation, and coagulation pathway activation. Key mechanistic insights reveal that endomysial fibrosis, rather than total fibrosis burden, specifically correlates with persistent atrial fibrillation and conduction abnormalities. Animal studies demonstrate that sustained atrial fibrillation over months results in progressive structural remodeling characterized by increased subepicardial fibrosis, leading to impaired transverse electrical conduction, endocardial-epicardial dissociation, and breakthrough wave emergence. Critically, a complete loss of anti-arrhythmic drug efficacy after prolonged atrial fibrillation establishes the mechanistic basis for treatment resistance. These findings suggest that fibrosis promotes both reentrant activity and enhanced automaticity through the disruption of electrical coupling and the creation of heterogeneous conduction patterns. Even after hemodynamic recovery, atrial fibrosis and conduction abnormalities persist, highlighting the largely irreversible nature of structural remodeling [12]. On the other hand, early recurrence of atrial tachyarrhythmia within three months following catheter ablation has been reported to vary between 35% and 50%, and approximately half of the patients did not experience a recurrence in the long-term follow-up period [13]. Consequently, early recurrence is usually managed conservatively. Premature recurrence could be linked to temporary factors, such as inflammation caused by ablation or effects on the autonomic nervous system. Nonetheless, it may also involve elements such as the reconnection of the ablation site or atrial fibrillation originating from non-pulmonary veins. In this study, the finding that early recurrence, even after initial improvement, was more frequently observed in the recurrent group 90 days or later after catheter ablation represents an essential observation for follow-up after catheter ablation [13]. Left atrial enlargement signifies structural remodeling and atrial fibrosis [14]. Previous research has demonstrated that low-voltage regions in the left atrium, which indicate atrial fibrosis, may elevate the risk of atrial fibrillation recurrence following ablation [15]. If early recurrence is caused by significant fibrosis in the left atrium, we consider the possibility that long-term recurrence may also be likely.

In this study, LAVI was significantly larger in the recurrent group than in the non-recurrent group. Regarding the relationship between atrial enlargement and atrial fibrillation, the impact of atrial fibrosis must be examined. The most important molecular mechanism of atrial fibrosis is the transforming growth factor (TGF)-β1/Smads signaling pathway [16]. Furthermore, it has been reported that fibroblast activation and abnormal extracellular matrix remodeling due to matrix metalloproteinases (MMP)/tissue inhibitor of metalloproteinase (TIMP) imbalance lead to widened gaps between cardiomyocytes, resulting in conduction delay, anisotropic conduction, and the formation of abnormal reentry circuits [17]. Additionally, electrical abnormalities due to changes in gap junction proteins and the progression of inflammatory fibrosis due to increased NLR family pyrin domain-containing 3 (NLRP3) inflammasome expression in atrial cardiomyocytes have also been suggested [18]. Future studies require evaluation using cardiac MRI and atrial biopsy. Previous reports indicate that atrial fibrosis is closely related to atrial volume and that increased atrial volume is a factor worsening atrial fibrillation and also a risk factor for recurrence after catheter ablation. We consider that these findings are consistent with the results of this study.

We demonstrated that additional LAPWI and CTI ablation were more common in the recurrent group than in the non-recurrent group. Although a previous study suggested that the recurrent rates of RFCA and cryoballoon ablation for paroxysmal atrial fibrillation are comparable [19], the present recurrent rate was significantly lower in cryoballoon than in RFCA (9% vs. 18%, P < 0.05) and in paroxysmal (10% vs. 15%, P < 0.05), persistent (6% vs. 13%, P < 0.05), and long-lasting persistent (17% vs. 25%, P < 0.05) atrial fibrillation. Additionally, LAPWI does not lead to an increase in recurrent rates for persistent atrial fibrillation [20]. This study did not perform causal analysis, but LAPWI and CTI ablation were frequently added to RFCA or cryoballoon ablation in the recurrence group, particularly in persistent or long-standing persistent atrial fibrillation. This suggests that the recurrent group after catheter ablation with persistent or long-standing persistent atrial fibrillation exhibited significant left atrial fibrosis and left atrial enlargement, making them challenging catheter ablation cases requiring the addition of LAPWI or CTI ablation.

We also found that decreased BNP levels and improved LVEF were observed in the non-recurrent group. Although exercise tolerance (NYHA classification) was not altered, this is thought to result from improvements in hemodynamics due to the elimination of atrial fibrillation and maintenance of sinus rhythm following catheter ablation, as well as systemic effects such as improvements in sympathetic nervous system activity and the renin-angiotensin system, leading to reduced afterload. In the following study, we must follow clinical outcomes and symptom improvements for an extended period. Moreover, the eGFR levels exhibited stability in the non-recurrent group, whereas a decline was observed in the recurrent group. The enhancement of renal function in individuals with atrial fibrillation and chronic kidney disease (CKD) following ablation therapy has been well-documented in prior research [21]. Nevertheless, the present study did not observe a similar outcome. On the contrary, declining renal function serves as a predictor of recurrent atrial fibrillation [22]. This assertion is substantiated by the association between atrial fibrillation and an increased risk of CKD, indicating a reciprocal relationship between CKD and atrial fibrillation [23]. The bidirectional association between atrial fibrillation and renal function suggests that declining renal function may be both a consequence and a contributing factor to the recurrence of atrial fibrillation. 

Early interventions aimed at rhythm control in cases of atrial fibrillation have been shown to improve prognosis [24]. Nevertheless, timely intervention is essential to prevent prolonged atrial fibrillation or increase LAVI to minimize recurrence post-catheter ablation. The results of this study suggest that prompt intervention plays a critical role in reducing the risk of recurrence after catheter ablation, primarily when implemented before the occurrence of prolonged atrial fibrillation or an elevation in LAVI. While observing a decrease in eGFR after one year in patients with atrial fibrillation who experienced recurrence, interventions focused on preserving renal function may decrease the probability of atrial fibrillation recurrence. We would need additional prospective studies in this particular context.

In this study, the recurrent group had a higher proportion of eGFR < 45 compared to the non-recurrent group. At one year, eGFR decreased in the recurrence group, whereas no significant decrease was observed in the non-recurrent group. Causal relationship analysis and whether eGFR decline constitutes a risk factor for recurrence could not be examined due to statistical limitations, as multivariate analysis was not performed. A previous report has suggested that prolonged catheter ablation procedure duration increases the risk of contrast-induced nephropathy and extends periods of hemodynamic instability, while intraoperative hypotension may cause a decrease in eGFR [22]. The present study may also detect this effect (prolonged procedure time). Impaired renal venous return by elevated left atrial pressure and increased inflammatory cytokines due to atrial fibrillation recurrence may also affect renal function [25]. However, this study did not collect the data necessary to investigate this possibility. Conversely, in the recurrent group, despite similar rates of hypertension and diabetes compared to the non-recurrent group, there were more cases of eGFR < 45, and although not significant, the mean values also tended to be lower. This suggests that a pre-existing state of worsening CKD (indicated by a larger eGFR slope) may be associated with a higher risk of atrial fibrillation recurrence. Meta-analysis papers have demonstrated that eGFR < 60 is associated with a significantly higher recurrence rate of atrial fibrillation after catheter ablation [26,27]. In CKD, atrial fibrosis is induced by activation of the TGFβ1/Smad2/3 pathway and NLRP3 inflammasome, as well as decreased connexin expression. Additionally, reduced K⁺ channel (Kv1.5) expression leads to prolonged action potential duration, decreased conduction velocity, shortened effective refractory period, and increased atrial conduction heterogeneity, which can trigger atrial fibrillation, and increased intra-atrial conduction heterogeneity, as reported in studies using CKD animal models [28,29]. Combined with our findings, the degree of eGFR decline may indicate recurrence risk after catheter ablation for atrial fibrillation. Therefore, patients with CKD at the time of ablation or those with a steep eGFR slope may require more careful follow-up. Furthermore, recent evidence suggests that, in addition to conventional blood pressure management with renin-angiotensin system inhibitors and calcium channel blockers, SGLT2 inhibitors may be beneficial as prevention for the recurrence of atrial fibrillation after catheter ablation for CKD [30]. We would need additional studies to assess the risk in this regard.

This study evaluated recurrence at one year after catheter ablation. However, the 2020 European Society of Cardiology (ESC) and 2023 American Heart Association (AHA) guidelines recommend treating atrial fibrillation as a chronic disease even after ablation, recommending regular long-term follow-up regardless of symptoms [1,9,10]. Even if atrial fibrillation has not recurred at one year, it has been reported that approximately 20%-25% of patients experience late recurrence within five years after the second year. This translates to an ongoing annual recurrence risk of about 4%-7%. Therefore, rather than concluding the evaluation after one year, it is recommended to continue with at least annual examinations and ECG testing thereafter. This study also necessitates the continuation of long-term evaluation.

The results of this study suggest that even with a certain level of operator proficiency at a core hospital in a regional area of Japan, it is necessary to always consider the possibility of a high recurrence risk at one year after catheter ablation in patients with long-standing persistent atrial fibrillation, early recurrence episodes, and elevated LAVI. Furthermore, if the eGFR has decreased one year after catheter ablation, it is necessary to explore the possibility of recurrence in greater detail. Additionally, during follow-up after catheter ablation, it is considered necessary to fully explain to patients with symptoms indicating a potential recurrence of atrial fibrillation that they should promptly seek medical attention.

In this study, no significant changes were observed in home blood pressure or heart rate during atrial fibrillation between the recurrent and non-recurrent groups. However, to examine the effects of sympathetic nerve activation, blood pressure and heart rate alone are insufficient; heart rate must also be considered in relation to oral medications affecting heart rate, such as beta-blockers. Future studies should evaluate sympathetic nerve activity using measures such as urinary catecholamines, heart rate variability, and myocardial scintigraphy.

Limitations of this retrospective follow-up study include the relatively small number of cases and the one-year follow-up period after catheter ablation. This study is a single-center retrospective analysis, making selection bias, measurement bias, and residual confounding factors unavoidable. Furthermore, it is impossible to completely rule out the possibility that differences in recurrence rates stem from patient characteristics specific to our institution, treatment methods, or operator skill. Consequently, these results cannot be generalized and merely describe the current situation at a single institution in Japan. Furthermore, due to insufficient sample size and data collection, accurate multivariate analysis could not be performed. Consequently, it was impossible to verify a causal relationship between ablation techniques and recurrence or draw definitive conclusions from this study's results. Additionally, the study did not analyze the effects of medication use, socioeconomic status, physical activity, or regional differences (diet, healthcare systems, genetic factors) on outcomes in hemodialysis patients. Consequently, this study was limited to describing differences in clinical backgrounds. Regarding treatment strategies, LAPWI and CTI ablation were primarily used for persistent or long-standing persistent atrial fibrillation; however, this study did not sufficiently evaluate the correlation between specific procedures and their efficacy. Furthermore, additional procedures were performed at the operator's discretion, which may have influenced the results. Asymptomatic AF recurrence could not be fully detected. To address these limitations, a multicenter prospective study with an adequate sample size is needed, employing a study design that enables statistically valid multivariate analysis, alongside a clinical system that ensures detailed data recording and database development. However, it is significant that all 368 patients were closely monitored at our institution and received comprehensive medical care.

## Conclusions

In conclusion, compared to the non-recurrent group, the recurrent group within one year after catheter ablation had longer procedure times and a larger LAVI. Early recurrence (within 90 days after catheter ablation) was also more common in the recurrent group. On the other hand, additional LAPWI and CTI ablation were more commonly chosen for the persistent and long-lasting persistent atrial fibrillation in the recurrent group. The non-recurrent group showed improved BNP, LAVI, and LVEF one year after the procedure. In the recurrent group, there was a notable decline in eGFR at one year after catheter ablation compared to that before catheter ablation. This study has many major limitations, as it is a retrospective study at a single regional core hospital in Japan, and we did not analyze recurrent risk or causality. However, in cases of catheter ablation for atrial fibrillation with significantly large LAVI (>49.8 mL/m^2^), eGFR < 45 mL/min/1.73 m^2^, extended procedure time, and early recurrence within 90 days after catheter ablation, recognizing the possibility of recurrence within one year after catheter ablation and conducting follow-up with a focus on renal protection may provide valuable insights for catheter ablation of atrial fibrillation in regional core hospitals in Japan.
